# Oligonucleotide-Based Approaches to Inhibit Dengue Virus Replication

**DOI:** 10.3390/molecules26040956

**Published:** 2021-02-11

**Authors:** Kingshuk Panda, Kalichamy Alagarasu, Deepti Parashar

**Affiliations:** Dengue & Chikungunya Group, ICMR-National Institute of Virology, 20-A, Dr. Ambedkar Road, Pune 411001, India; kingshukpanda7@gmail.com

**Keywords:** antiviral, dengue, RNA interference, ribozymes, antisense oligonucleotides

## Abstract

Dengue fever is one of the most common viral infections affecting humans. It is an expanding public health problem, particularly in tropical and subtropical regions. No effective vaccine or antiviral therapies against Dengue virus (DENV) infection are available. Therefore, there is a strong need to develop safe and effective therapeutic strategies that can reduce the burden and duration of hospitalizations due to this life-threatening disease. Oligonucleotide-based strategies are considered as an attractive means of inhibiting viral replication since oligonucleotides can be designed to interact with any viral RNA, provided its sequence is known. The resultant targeted destruction of viral RNA interferes with viral replication without inducing any adverse effects on cellular processes. In this review, we elaborate the ribozymes, RNA interference, CRISPR, aptamer and morpholino strategies for the inhibition of DENV replication and discuss the challenges involved in utilizing such approaches.

## 1. Introduction

**Figure 1:**
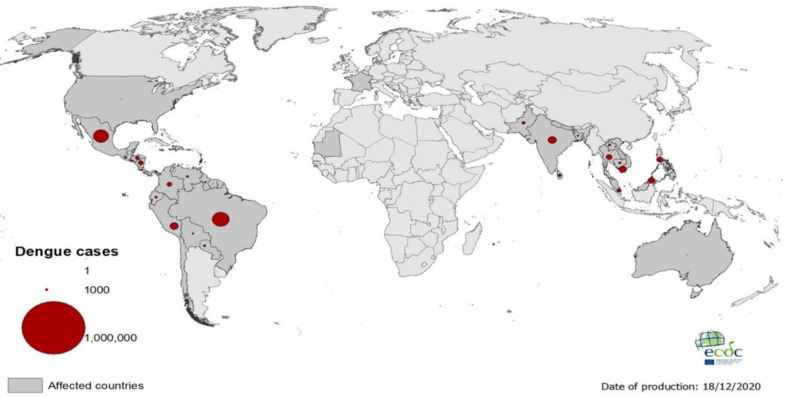
The global map for the prevalence of Dengue fever as of 18 December 2020 (Source: European Centre for Disease Prevention and Control).

**Figure 2:**
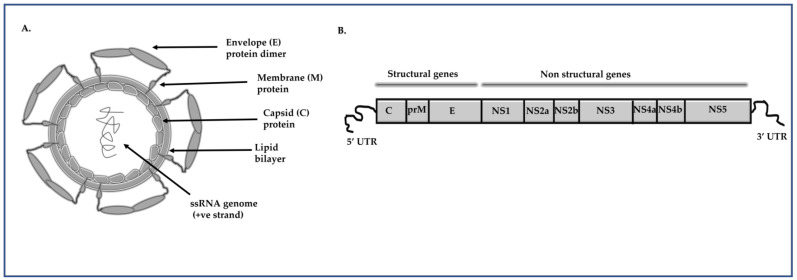
The Dengue virus (DENV) structure and genome. (**A**) General structure of DENV. (**B**) Genomic organization of DENV.

Dengue virus (DENV) is an important mosquito-borne viral pathogen known to cause dengue and severe dengue. An annual incidence of approximately 300 million DENV infections has been estimated and the disease burden is significantly higher in tropical and subtropical regions (Figure 1) [1]. It has been estimated to cause a global economic burden of USD 8.9 billion per year [2]. DENV has a positive-sense single stranded RNA genome, approximately 11 kb in size, which codes for three structural proteins including Capsid (C), pre membrane (prM) and envelope (E) proteins, and seven non-structural (NS) proteins including NS1, NS2A, NS2B, NS3, NS4A, NS4B and NS5 (Figure 2) [3]. The genome is translated as a single long polypeptide chain and later processed into separate proteins through viral and cellular proteases [4]. There are four different serotypes of DENV, DENV-(1–4), and the mechanism of attaching to the cell receptor is identical for all serotypes. DENV mainly attaches to receptors such as CD14, GRP78/bipheperansulphate, high affinity laminin receptor, and dendritic cell (DC)-specific intercellular adhesion molecule-3-grabbing nonintegrin (DC-SIGN) to enter into the cell by making conformational change in the E protein [5]. The internalization of DENV occurs through a pH-dependent receptor mediating the endocytosis process [6]. Patients infected with DENV can develop either mild or severe disease, including the occasionally life-threatening severe dengue. The non-severe dengue cases are further categorized into those with dengue without warning signs and those with dengue with warning signs. Major warning signs generally reported for dengue are abdominal pain, persistent vomiting, fluid accumulation, mucosal bleeding, lethargy, restlessness, liver enlargement, and an increase in platelet count [7]. Severe dengue is characterized by the presence of any of these abnormalities, such as severe bleeding, organ impairment, and/or plasma leakage [8]. Severe dengue is generally characterized by plasma leakage from blood vessels, and when plasma leakage is severe, it leads to shock and impairment of organs [9]. Altered immunity due to cross-reactive T cells and non-neutralizing antibodies produced during secondary infection with a heterologous DENV serotype results in an inflammatory cytokine storm and contributes to severe dengue [10]. The updated DENV treatment guidelines published by the WHO reports two main aspects of treatment: symptom specific therapy and fluid administration [11]. Currently, there is no specific antiviral available for treating DENV infection except for patient supportive care [12]. In certain countries, there is a vaccine named Dengvaxia®, which is approved for use in subjects with previous exposure to DENV [13]. Two other chimeric vaccine candidates are currently under Phase III trial; one developed by Takeda (TAK-003) and the other by the National Institute of Allergy and Infectious Diseases (TV003/TV005) [14,15]. Different approaches, such as structure-based computational docking studies on DENV proteins, followed by function-based in vitro assays, have been used for identifying antivirals against DENV [16,17].

Drug repurposing approaches are also being attempted against DENV. Previously approved drugs such as chloroquine, celgosivir, ribavirin, prednisolone, lovastatin underwent clinical trials, but none of these showed promising activity against dengue [18,19]. Inhibitors of dengue structural and non-structural proteins have also been reported. Medicinal plants with potent antiviral activity against DENV have also been identified. Traditional medicines are used in Asian and African countries which are mainly based on knowledge, experience, and practices [20].

Despite intense research efforts, finding an effective antiviral agent is elusive and an effective strategy for treating dengue is urgently needed. Targeting the viral proteins for antiviral activity is the major convention used for identifying antiviral agents. Although, targeting the viral genome to inhibit replication of the virus is an attractive strategy which can be employed against different viruses. Oligonucleotides are generally employed to target the viral genomes, resulting in inhibition of replication and or translation, also known as silencing of the viral genome expression. In this review, we discuss the different oligonucleotide-based approaches employed/available for targeting dengue.

## 2. Oligonucleotide-Based Approaches to Inhibit DENV Replication

These nucleic acid-based agents generally interact with a specific region of the target genome and downregulate the gene expression or completely disrupt its normal expression. Oligonucleotide-based approaches use different types of RNA or DNA molecules such as ribozyme, siRNA, miRNA, CRISPR, aptamer and morpholino to silence the expression of the viral genome.

### 2.1. Ribozyme

Ribozymes are small catalytic RNA molecules which are targeted towards RNA for cleaving at a particular site [21]. In 1982, Tom Cech and his research team at the University of Colorado first discovered the ribozymes or catalytic RNAs that were capable of excising themselves under in vitro condition [22]. Catalytic RNAs are of two types, based on the size: large catalytic RNAs and small catalytic RNAs. Large catalytic RNAs include group I introns, group II introns, and RNase P. Small catalytic RNAs include hammerhead ribozyme, hairpin ribozyme, hepatitis deltoid virus ribozyme and Varkud satellite (VS) ribozyme. Except for RNase P, all other ribozymes catalyze reactions that alter themselves [22]. However, these catalytic RNases can be modified to cleave other RNA molecules.

#### 2.1.1. Ribozyme Mechanism

**Figure 3:**
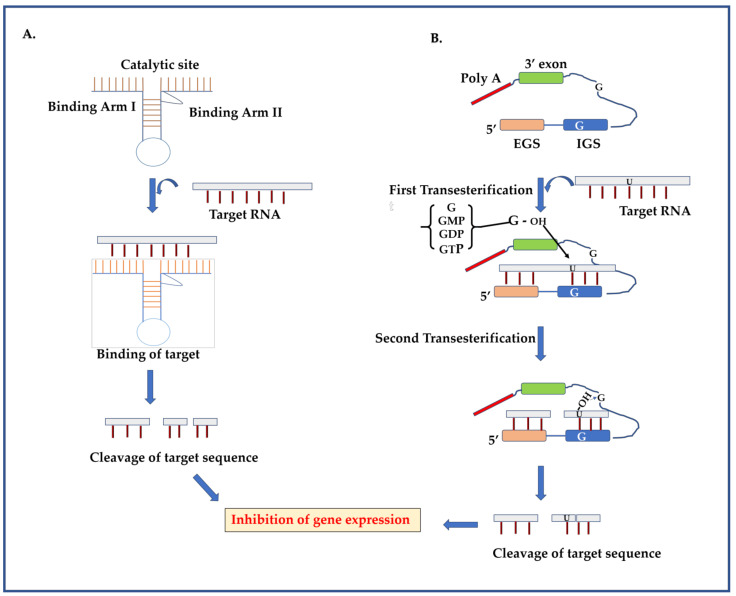
Pathway of gene silencing by the ribozyme-based strategy. (**A**) Hammerhead ribozyme has two binding arms and one catalytic site. The binding arm binds with the target sequence by Watson-Crick base pairing and makes a ribozyme-target complex. The catalytic domain cleaves the target region into small segments and as a result, inhibition of gene expression occurs. (**B**) Group I introns catalyze the trans-splicing reaction. During the first transesterification reaction, –OH group of free-floating guanosines (G/GMP/GDP/GTP) attack phosphodiester backbone which is directly downstream of reactive uracil. The 3′ exon then gets positioned at a close proximity towards the newly freed 3′–OH. This 3′–OH group attacks the phosphodiester backbone of group I intron, specifically, just near the upstream of 3′exon. The end result is catalytic cleavage of target RNA into small segments.

Hammerhead and group-I intron ribozymes are the most promising in inhibiting viral replication due to their strong binding efficacy and catalytic activity. The hammerhead ribozymes (hhRz) have a length of 40–50 nucleotides forming two domains; a functional domain and a structural domain (Figure 3A). The structural domain is associated with target specificity, whereas the functional domain is involved in the catalytic activity [23]. The functional catalytic center comprises 15 nucleotides which are highly conserved and surrounded by three double helices (I–III). Several hydrogen bonds along with multiple magnesium ions are present in the active site and helps in the cleavage reaction [24]. Ribozyme initially acts as an antisense RNA molecule and forms a partially duplex RNA structure with the target sequence. In the second step, the catalytic ability of ribozyme cleaves the target sequence through the mechanism of site-specific hydrolysis and release of the cleavage product. The cleavage occurs at a site near the NUH triplet wherein N is any nucleotide, while H represents all nucleotides except guanosine [25]. 

The Group I intron ribozymes have recently been in focus for therapeutic development due to their strong catalytic activity to cleave either single stranded or homologouslypaired double stranded RNA at a defined base [26]. The group I introns have four conserved short sequence elements, known as P, Q R, and S, and can fold into ten paired segments (P1–P10), among which P3, P4, P6 and P10 form the catalytic core. The group I intron catalyzes two reactions to excise them from the precursor (Figure 3B). Group I introns requires an accessible uracil nucleotide at downstream of the target sequence cleave point. The internal guide sequence, a short portion of P1 segment and an external guide sequence, helps in target binding. In the first step, the group I intron completes one catalytic step by separating a 5′exon from the intron using a 3′ hydroxyl of an exogenous guanosine at the 5′ splice site. The 3′hydroxyl of the free 5′exon attacks the 3′ splice site to release the intron, which is a circular product that retains the catalytic activity and substrate specificity [22]. The following mechanism makes ribozyme an efficient antiviral technology against several viruses, including DENV. 

#### 2.1.2. Targeting DENV Genome Using Ribozymes

Nawtaisong et al. [27] designed 14 hammerhead ribozymes (hhRz) which were expressed using pan retroviral vectors under the control of *Aedes aegypti* tRNA^val^ promoter for suppressing DENV-2 replication in lentivirus-transduced C6/36 mosquito cells. These hhRz also contained 3′polyA tail of 6o bases length which helped in cleaving the target RNA within the secondary structures. These hhRz RNA cleaved the targets at NUH triplet site. In C6/36 cells transduced with pantropic retroviral vectors expressing certain hhRz RNA, 100-fold suppression of DENV-2 replication was observed. Carter et al. [28] designed group I trans-splicing introns targeting two different uracil bases in the conserved 5′-3′ cyclization sequence (CS) region which is common to all serotypes of DENV. In *Aedes aegypti* cell lines stably expressing these group I trans-splicing introns, suppression of DENV-2 virion production was observed compared to non-transformed cell lines. Though anti DENV group I introns can suppress the virus replication in mosquito cells, this alone is not sufficient to prevent the emergence of escape mutants of DENV. Later, the research group coupled the splicing activity of group I intron with death-upon-infection strategy [29]. In this strategy, the anti DENV group I intron was coupled with an apoptosis inducing gene ΔN Bax 3′ exon. This gene lacks the Bax-H3 domain which interacts with anti-apoptosis regulators and hence can induce apoptosis irreversibly. In mosquito cell lines transformed with the vector containing anti-dengue group I intron and ΔN Bax 3′ exon, enhanced antiviral activity was observed and due to the apoptotic activity of the ΔN Bax 3′ exon, this inhibition of DENV does not require cleavage of all DENV genome by group I introns. They also designed dual acting group I introns coupled with ΔN Bax 3′ exon which can target both DENV and chikungunya virus and demonstrated inhibition of replication of both the viruses in transformed mosquito cells. All these reports suggest that designed ribozymes were active against DENV serotypes and showed a significant reduction in viral RNA although in vitro studies using human cells and in vivo studies using animal models are required [30]. 

### 2.2. RNA Interference

RNA interference is an evolutionary mechanism of gene regulation that is induced by small RNA molecules along with a specific endonuclease. In the year 1990, Napoli and Jorgensen [31] first reported the phenomenon of RNA interference during the study of chalcone synthase (CHS) as a rate limiting enzyme during anthocyanin biosynthesis in plants. In animals, this phenomenon was first documented by Guo and Kemphues [32], who observed the association of sense and antisense strand during *Caenorhabditis elegans* embryo development. Small interfering RNA (siRNA) and microRNA (miRNA) are the two main categories of small RNAs which are being investigated extensively for their multiple roles in regulating gene expression and antimicrobial activity. siRNAs and miRNAs have emerged as practically modular and adaptable therapeutics for treating viral infections [33].

#### 2.2.1. RNA Interference Mechanism

**Figure 4:**
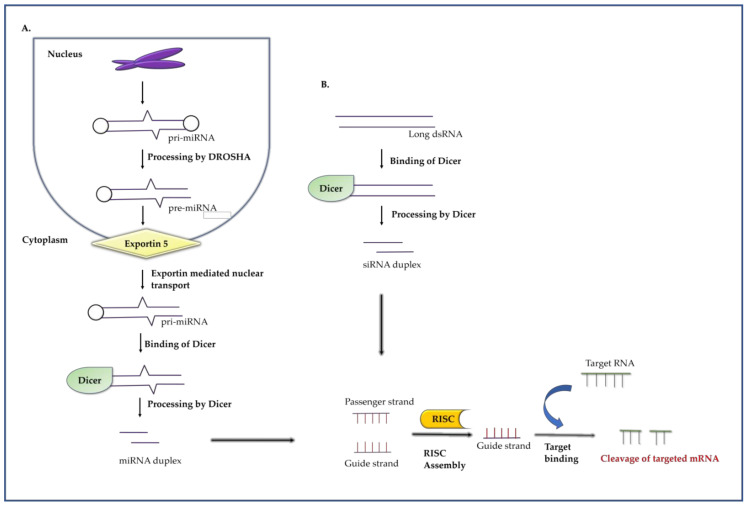
Gene silencing mechanism of RNA interference. (**A**). The pri-miRNA is processed into pre-miRNA with the help of enzyme DROSHA during the first step of miRNA mediated gene silencing. The pre-miRNA is transported into the cytoplasm from nucleus with the help of an enzyme named exportin-5. The enzyme Dicer processed the pre-miRNA into miRNA duplex. The miRNA duplex then associates with RISC complex and cleaves the targeted mRNA. (**B**). Long dsRNA generates small siRNA duplex by the action of DICER. The RISC loaded onto the duplex and the complex binds with the target complementary strand. The mRNA strand is now cleaved through endogenous mechanism of AGO2 endonuclease present in RISC complex.

Both siRNA and miRNA exert gene silencing in a post transcriptional manner [34]. siRNA can target and cleave a single specific mRNA while miRNA can target multiple mRNA [34]. The first step of RNA interference mechanism is the entry/release of dsRNA into the cytoplasm of a cell [35]. The microRNA (miRNA) gene transcription is carried out in the nucleus and transcribed as a pri-miRNA by RNA polymerase II enzyme. The pri-miRNA is processed into 70–100 nt-long hairpin pre-miRNA by a microprocessor complex, comprising of a RNase III enzyme, Drosha, and a dsRNA binding protein, DiGeorge syndrome chromosomal region 8 (DCGR8). The pre-miRNA then gets transported to the cytoplasm from nucleus with the help of an enzyme named exportin-5 and it is further processed into miRNA duplex of 18–25 nucleotides by the enzyme Dicer. The miRNA duplex then associates with RNA-induced silencing complex (RISC) complex which is formed by the incorporation of short miRNA in to an argonaute-2 (AGO2)’ endonuclease containing silencing complex. Finally, miRNA-RISC complex cleaves the targeted mRNA through complementary base pairing and endonuclease activity (Figure 4A).

In the siRNA-mediated RNAi pathway, dsRNA is processed into 21–30 nt-long short siRNA molecules that act as a module of silencing mechanism. The silencing pathway involves chopping of dsRNA into siRNA by a RNase III family of enzyme, known as “Dicer” and introduce two nucleotide overhangs at the 3′ ends. Dicer has four distinct domains: an RNase/Helicase domain, dual catalytic RNase III domain, a double stranded RNA binding domain and a PAZ domain [36]. Short siRNA is now incorporated into an AGO2 endonuclease containing silencing complex to form a RISC. Now the RISC is activated, and it provides target specificity by unwinding two siRNA strands and target the complementary mRNA by base pairing with the guide strand. The mRNA strand complementary to the bound siRNA is now destabilized and degraded through endogenous mechanism of AGO2 endonuclease (Figure 4B). Instead of dsRNA, synthetic siRNA can also be utilized, which does not require chopping by Dicer [37].

#### 2.2.2. RNAi as an Antiviral against DENV

Zhang et al. [38] first reported the designing of potential siRNAs which can inhibit DENV replication in both Vero cells and human DCs. They developed an adenovirus mediated siRNA cassette which target the 3′ cyclization sequence (CS) region of dengue (1–4) genome. Later, researchers developed a DC-targeted siRNA delivery mechanism to suppress DENV infection and associated proinflammatory cytokine production in both in vitro and in vivo system [39]. They demonstrated that DC3-9dR chimeric peptide is able to deliver a potent siRNA which targeted the cd loop-coding sequence within the domain II of the DENV-2 envelope protein.

The RNA interference mediated by siRNAs is now being widely used to knock down several important viral genes in a sequence-specific manner. A study reported the designing of four siRNAs targeting DENV-1 viral membrane glycoprotein precursor (prM) gene [40]. The study found that one siRNA reduced the viral RNA about 40-fold with an inhibition rate of 97.54%. A synthetic DC-3 siRNA targeted towards the highly conserved 5′ 5′CS region of four DENV genome has been reported to exert maximum reduction in DENV infection in the Huh7 cell line and AG29 mice [41]. The identification of functional gene is an important criterion for siRNA-specific therapeutics. Some reports are available regarding the detailed profile of host genes which are important for DENV cellular entry [42]. siRNAs designed against the GRP78 host cell receptor can inhibit DENV attachment and clathrin-mediated endocytosis in the HepG2 cells and have been reported to cause significant reduction in both viral load as well as the number of DENV virus-infected cells [43]. Conserved sequences within the NS4B and NS5 gene have been targeted for designing siRNAs. Three first generation siRNAs have been identified to inhibit the replication of DENV-1, -3 and -4 in Vero and C6/36 cells, while a second generation siRNA targeting NS5 has been reported to inhibit DENV-2 replication in both mammalian and mosquito cell lines [44,45].

Apart from siRNA, few studies have reported the inhibition of DENV by using miRNAs. miR-252 from *Aedes albopictus* has been shown to interact with the E glycoprotein of DENV and influence its expression. Inhibition of miR-252 in C6/36 cell line led to an increased expression of DENV-2 E protein and DENV-2 RNA copies while, over expression of miR-252 reduced DENV-2 RNA copies and the expression of viral E protein [46]. Another study identified a miRNA named miR-548 g-3p, which was able to interact with stem loop A (SLA) promoter present in the 5′UTR region of all DENV serotypes [47]. A significant reduction in viral load for all DENV serotypes was observed as the miRNA inhibited the interaction between SLA and NS5 promoter leading to inhibition of replication. A synthetic miRNA, miRNA133a, has been shown to directly bind to the 3′SL loop of the 3′UTR and suppress DENV replication in Vero cells [48]. Later, Betanchur and Inchima [49] designed two miRNAs against all DENV serotypes through bioinformatics approaches and tested experimentally in Vero cells. They proposed that the designed siRNAs-miR-484 and miR-784 could downregulate the DENV replication in a similar manner like miRNA133a.

Though siRNAs can effectively inhibit the replication of DENV in both in vitro and in vivo, to date, there have been no in vivo studies regarding the antiviral activity of miRNA against DENV.

### 2.3. Aptamer

Aptamers are a class of oligonucleotides (either DNA or RNA) which can bind specifically with a protein by three-dimensional conformation. Aptamers can be considered as an alternative to antibodies. In 1990, Jack Szostack demonstrated the isolation of a short RNA oligonucleotides which were able to interact with organic dyes and coined the term “Aptamer” [50]. In the same year, Larry Gold and his team reported about an efficient strategy for selection of aptamer that is known as “systemic evaluation of ligands by exponential enrichment (SELEX)” [51]. SELEX is a process which is used for generating target-specific aptamers against any target region. Initially, the aptamers were generated through SELEX by constructing a library of 5′ or 3′ flanked regions which serves as a primer sequence [52]. Both DNA and RNA aptamers showed great advantages in the emerging field of molecular virology as they can act as both antivirals and diagnostic tools [53]. In recent years, aptamers have received strong focus as therapeutics and the US Food and Drug Administration has approved an aptamer to treat macular degeneration of eye [52].

#### 2.3.1. Mechanism of Aptamer and Identification of Target Specific Aptamers

**Figure 5:**
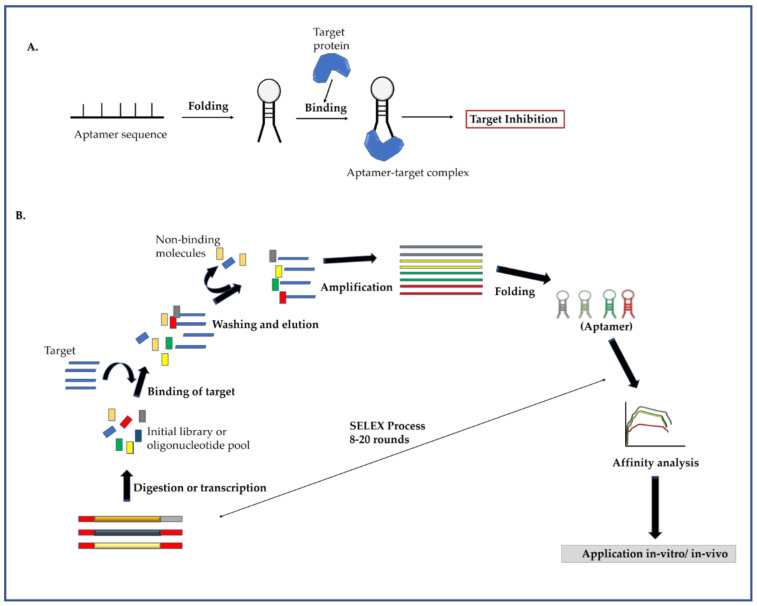
Aptamer as an antiviral. (**A**) The diagram showing schematic representation of aptamer binding towards a target. The aptamer folded itself in a 3D configuration after adjustment in a suitable condition. The target binds with the folded aptamer and forms an aptamer target complex and resulting in target inhibition. (**B**) Schematic illustration of aptamer selection by SELEX technology. The screening cycle starts with incubating target molecules with the oligonucleotide pool. After washing of non-binding molecules, the bound molecules were amplified and analyzed for binding affinity. The cycle continues for 8–20 rounds for generating an effective aptamer library.

Aptamers are short DNA/RNA sequences which can fold into a complex three-dimensional structure and bind towards a targeted strand (Figure 5A). The interaction between the aptamer and target depends on the three-dimensional folding pattern which is generated due to intermolecular hybridization of the oligonucleotides. The aptamers are also termed as “synthetic antibodies”, as they can bind towards the target or antigen in a similar pattern-like antibody [54]. RNA aptamers are generally used for designing therapeutic agents, whereas DNA aptamers are widely used for diagnostic purpose as an alternative to antibodies. Aptamers have been prepared and reported against a wide range of viral proteins. The selection of aptamers follows a common SELEX strategy (Figure 5B) which helps in finding nucleotide molecules with high affinity towards a target molecule. The SELEX techniques comprising of three major principles: binding of antisense nucleotides, removal of unbound nucleotide and amplification of selected nucleotides [55]. In the first step, an oligonucleotide library is generated which contains a central randomized region flanked by anchor sequences to allow PCR reaction.

The library is generated for providing different confirmation variabilities of oligonucleotides towards a target sequence. In the second step, the oligonucleotide pool is incubated with the target molecule and selected based on interaction ability. The unbound oligonucleotides are removed from the reaction setup, as those are not able to interact and contain less binding capability. In the final step, the recovered aptamer or oligonucleotides are PCR amplified to generate a pool of high affinity aptamers with less variability. Maximum rounds of SELEX are generally performed to obtain an aptamer population with the highest binding affinity towards the target. The aptamers are flexible and can be further modified to improve their physical and chemical appearance such as stability, nucleophile attack escape, and others [56].

#### 2.3.2. Inhibition of DENV Replication by Aptamer

The antibody-dependent enhancement (ADE) mechanism is a special immunological phenomenon which is generally associated with secondary DENV infections. During secondary infection with a heterologous serotype, non-neutralizing, cross reactive antibodies are produced against the prM protein and these antibodies enhances the infection of DENV by binding to Fc receptors on the monocytes/macrophages. This phenomenon is known as ADE and is considered as one of the major contributors to disease severity [57]. Due to ADE, no effective antibody against DENV has been approved for clinical treatment. The hypothesis of disrupting DENV capsid protein and host protein interaction by using aptamers was first reported in 2013 [58]. DENV C protein interacts with the multifunctional host protein nucleolin (NCL), which is important for viral replication cycle inside the host. The study showed that disruption of this interaction by NCL-binding aptamer (AS1411) can reduce the viral load in DENV infected cells and it can be a promising approach for the development of antiviral against DENV. The designing of potential aptamer against all serotypes of DENV was reported in 2015 [59]. The designed aptamer S15 binds to the E protein domain 3 (ED3) and neutralizes the DENV. The authors concluded that the smaller size of the aptamer helps to interact with target region of four different dengue serotypes more efficiently and also possesses strong neutralization activity than cross-reactive antibodies. Later, Jung et al. [60] designed RNase-resistant 2′-fluoro-modified RNA aptamers against the methyl transferase (MTases) domain of DENV-2 and -3. The study concluded that RNase-resistant 45-mer truncated aptamers competitively inhibited the binding of genomic RNA towards the MTases and *N*-7-5′ capping. The aptamers can be promising therapeutic agents due to high efficacy, low toxicity, strong pharmacokinetics mechanism and low cost.

### 2.4. Morpholinos

Morpholinos are a synthetic antisense oligonucleotide which can bind to a target mRNA by Watson-Crick base pairing and block the translation through RNase H-independent steric blockade. In 1985, Summerton came up with a morpholino structure which showed strong pharmaceutical properties and therapeutics applications [61]. The morpholino antisense oligos were initially designed as a custom research agent for gene activation/deactivation in developmental biology studies. The morpholino-based therapy has been evaluated for several genetic disorders, cancers, bacterial infections, and viral infections. A morpholino-based drug EXONDYS 51^TM^ (Eteplirsen) has already been approved by the U.S. Food and Drug Administration for the treatment of the genetic disorder, Duchenne muscular dystrophy (DMD) [62]. In recent years, researchers have been focusing on the morpholino-based therapeutics for DENV infections.

#### 2.4.1. Mechanism of Morpholino

**Figure 6:**
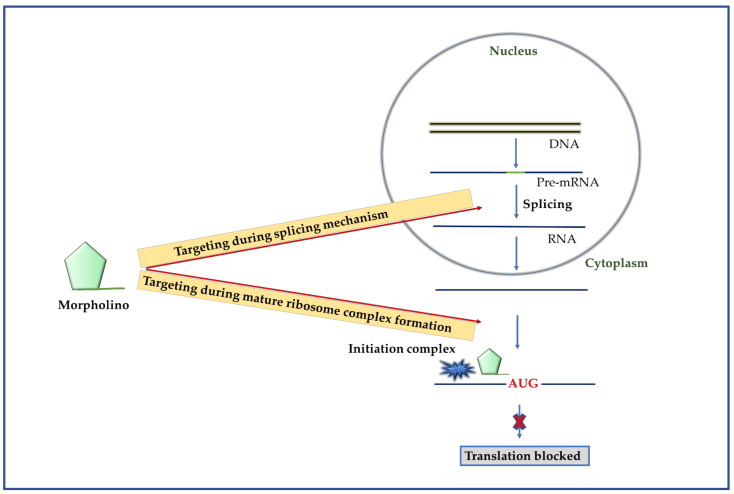
Morpholino as an antiviral. (**left**). The general structure of a morpholino phosphoroamidate. (**right**). Pathway of gene silencing by morpholino. Morpholino can block the mRNA maturation during splicing mechanism within the nucleus. It can also block the translation initiation complex binding site on mRNA and prevent the translation event.

The morpholinos are non-ionic in nature and possesses a backbone composed of morpholine rings and phosphonodiamidite inter-subunit linkages. The most common type of morpholino possessing a phosphorodiamidate backbone is known as phosphorodiamidate morpholino (PMO). The PMO (Figure 6A) was synthesized by substituting a morpholino moiety in the ribose ring and replacing the phosphodiester inter-subunit bonds with a phosphorodiamidate linkages [63]. The unmodified PMO had faced several delivery problems in animal models, as it requires additional delivery reagents and low-serum conditions [64]. Thus, to overcome this issue, two other forms of PMOs with chemical modifications were developed, including peptide-conjugated PMO (PPMO) and Vivo-PMO [65].

Once morpholino is inside the cell, it covers the mRNA target sequence like masking tape. The typical translation machinery starts with the binding of small ribosomal subunit within the 5′ UTR region and starts the formation of the initiation complex (Figure 6B). The steric blocking of morpholino stops the formation of initiation complex for protein translation and, as a result, the translation machinery cannot bind the start codon. In order to prevent translation, morpholino must interrupt the process before the formation of the mature ribosome complex, as the mature ribosome can displace bound morpholino. The morpholinos are also reported to block the splicing mechanism of mRNA when it binds to the exon or intron boundary.

#### 2.4.2. Targeting DENV Using Morpholino

Kinney et al. [66] first designed and tested a panel of peptide-conjugated phosphorodiamidate morpholino (PPMO) against DENV-2. The study reported that PPMO targeted towards the 5′-terminal nucleotide (5′SL) and 3′ cyclization sequence (3′CS) regions of DENV-2 showed maximum reduction in the viral titer in comparison with the control. PPMO targeted towards 3′-terminal nucleotides and AUG translation start site region or 5′ cyclization sequence region of DENV-2 showed relatively poor or moderate suppression, respectively. Interestingly, the study also reported that a particular concentration (10µM) of 3′CS targeted PPMO inhibited the replication of all four serotypes of DENV. Holden et al. [67] designed a third PPMO targeted towards the 3′ stem-loop (3′SLT) of DENV and tested it in BHK cell line. The results reveal that 3′SLT-targeted PPMO inhibited DENV replication by interfering with both mRNA transcription and protein translation machinery. The 5′SL PPMO inhibited viral replication by interfering with the translation process and 3′CS blocked viral RNA synthesis whereas 3′SLT act by both way which was found to be unique. An in vivo study of peptide-conjugated phosphorodiamidate morpholino (PPMO) revealed the antiviral efficacy in both pre-treatment and post-treatment conditions [68]. Anti DENV PPMO against the 5′SL and 3′CS region was designed and injected into the AG129 mice model in a dose-dependent manner. The study reported that PPMO pre-treatment helped in extending the average survival time up to 8 days with a little toxic impact on overall mouse health.

Vivo morpholino has also been reported to be a potent antiviral against DENV which can bind to the RNA and can penetrate directly into the cells [69]. The first vivo-morpholinos against DENV targeted the 3′SL portion of DENV 3′UTR region and was tested in Vero, A549, U937, and human monocyte cell lines. The result show that the vivo-morpholino can bind to the DENV RNA target sequence and inhibit virus production in all cell lines by more than 10^4^-fold when compared with untreated cells at a concentration of 4 µM. This group also reported the inhibition of DENV in monocyte-derived dendritic cells (MDDCs) by using vivo-MO-1 targeting 3′SL of DENV [70].

## 3. Other Oligonucleotide-Based Methods Reported for DENV Inhibition

Clustered regularly interspaced short palindromic repeats (CRISPR)-based antiviral technology is also currently in focus to treat DENV infection. During the last few years, CRISPR technology has introduced itself as an effective antiviral therapeutic strategy against several viral pathogens. The Type II and VI CRISPR/Cas system, especially for Cas 9 and Cas 13, is the most extensively characterized system which has been reported to exert strong antiviral activity.

The life cycle of flaviviruses is complex and relies on host factors. CRISPR-based genetic screens have been used to identify the target proteins that are essential for DENV replication. SPCS 1 proteins, AXL receptor tyrosine kinase (AXL), RAB5C, RABGEF, N-deacetylase and *N*-sulfotransferase 1 (NDST1), exostosin glycosyl transferase 1 (EXT1) and endoplasmic reticular membrane complex (EMC), and oligosaccharyltransferase have been identified to be important in different stages of the DENV life cycle through CRISPR-based genetic screens [71,72,73,74]. A recent study showed that CRISPR Cas13a protein is capable of knocking down RNA expression of DENV effectively in mammalian cells [75]. A CRISPR RNA targeting the NS3 gene effectively inhibited DENV in Vero cells [75].

Properties of different oligonucleotides based antiviral approaches against DENV have been summarized in Table 1.

## 4. Challenges Associated with Oligonucleotides-Based Antiviral Methods and Possible Ways to Overcome the Hurdles

Theoretically, oligonucleotides can be designed and targeted towards any gene of interest, which makes them an efficient tool for a broad range of applications. Although oligonucleotide approaches have advantages, they have faced many hurdles during clinical applications. Localized and inefficient delivery of oligonucleotides is the major drawback noticed for all the approaches. Due to their uncharged nature, oligonucleotides such as morpholino face poor cellular uptake and rapid clearance [113]. Interestingly, recent findings reported new target specific delivery methods such as viral vector-associated delivery, liposome-mediated delivery, and nanoparticle-mediated delivery. Pieve et al. [114] reported a novel approach of conjugating the aptamer to a synthetic polymer, polyethylene glycol (PEG), which helped in increasing the aptamer half-life and resulted in higher cellular uptake. Secondly, finding the suitable target site is also important for oligonucleotide-based silencing approaches. Bioinformatics-based and computational approaches are generally used for identifying target genes and to reduce the chance of viral escape. The off-target activity of designed oligonucleotides also needs to be minimized to ensure safety in an in vivo application. Thirdly, the development of viral resistance towards any oligonucleotide-based antiviral techniques can generate a more deadly or pathogenic viral mutant. This is a major concern and needs to be addressed before clinical practice. During cleavage of the viral genome, small segments of the viral genome are produced. Complete elimination of these short segments of the genome is required for successful inhibition as these small segments can show antigenicity. PMO-based resistance has been reported for two mutant strains of West Nile virus and Ebola virus [115,116]. Therefore, strategies such as the incorporation of promiscuous bases, indirect inhibition of host factor required for virus replication and careful selection of target sequence must be explored to avoid the emergence of resistance viruses. Finally, synthetic oligonucleotides such as aptamers have low stability in biological fluids and are highly prone to nuclease degradation. Recent studies have shown that chemical modifications in the sugar-phosphate backbone of aptamers increased their stability and protected them from degradation [117,118,119,120]. Non-natural alternatives to nucleic acids, also known as xenonucleicacids (XNA), have been reported to be useful in designing nuclease-resistant aptamers [121]. A 2-deoxy-2-fluoroarabino nucleic acid (FANA)-based aptamer targeting HIV-1 reverse transcriptase, a 2-*O*-methyl-ribose–1,5-anhydrohexitol nucleic acid (MeORNA–HNA) aptamer targeting the rat vascular endothelial growth factor 164, and a threose nucleic acid (TNA) aptamer targeting human thrombin have also been reported [119,121]. Such XNA-based aptamers might be useful for inhibiting DENV. Moreover, immune response and possible side effects in the host should be investigated, since oligonucleotides can elicit immune response by activating toll like receptors.

## 5. Conclusions

The first licensed DENV vaccine Dengvaxia® led to considerable controversy worldwide due to major limitations [14]. It is challenging to develop a safe and effective antiviral compound towards DENV. The use of oligonucleotides is considered as a very attractive means of inhibiting viral replication. A major advantage is the relatively simple rational design of oligonucleotides which should bind only to specific nucleic acid sequences or protein targets. Low molecular weight of oligonucleotides also makes it a valuable alternative for antivirals. The number of studies on oligonucleotide-based antivirals against DENV has shown an increasing trend in recent years, but the technology has experienced many ups and down. Oligonucleotide-based technologies have shown antiviral properties with low toxicity in both in vitro and in vivo studies, providing an initial indication towards the future pharmaceutical developments. These issues are being investigated to find solutions which will help to move forward towards the era of oligonucleotide-based therapeutics against DENV infection.

## Figures and Tables

**Table 1:** An overview of key differences between different oligonucleotide-based antiviral approaches against DENV.

	Ribozyme	RNA Interference	Aptamer	Morpholino
**Mode of Action**	Chemical catalysis reaction [76]	RISC mediated breakage [77]	Intermolecular hybridization-based inhibition [78]	Steric blocking of translation [79]
**Structural Components**	Functional domain and structural domain [80,81]	Guide strand and Passenger strand [82]	Small ss DNA, RNA or synthetic XNA [83]	Nucleotide bases attached with methylenemorpholine rings/oligonucleotide analogs [84]
**Specificity**	High [85]	High [82]	High [86]	High [87]
**Off Target Effects**	Limited [88]	Extensive [89]	Limited [90]	Extensive [91]
**Target site**	3–10 nt [88]	18–20 nt [92]	30–100 nt [93]	5′UTR-first 25 nt [94]
**Recognition Mechanism**	Watson-crick base pairing [95]	Watson-crick base pairing [96]	Similar like antigen-antibody interaction [97]	Watson-crick base pairing [98]
**Required Sequence Information**	Transcriptome [21]	Transcriptome [77]	Transcriptome/Transcription factor [99]	Transcription start site (TSS) [100]
**Delivery**	Direct delivery, viral vector mediated [101]	Bathing, feeding, injection, transfection, transduction, transgenic [102]	Viral vector, carrier mediated [103]	Lipid-based delivery system [79]
**Time to Effect**	May require a week [88]	2–3 days [88]	Several days [104]	4–6 days [94]
**Cytotoxicity**	High [105]	Variable to high [106]	Non-Toxic [104]	Variable to high [105]
**Cost**	Low [107]	Low [108]	Low [83]	High [91]
**Immunogenicity**	Moderate [109]	High [110]	Less [111]	Less [112]

## Data Availability

Data sharing not applicable. No new data were created or analyzed in this study. Data sharing is not applicable to this article.

## References

[1] Cuypers: L., Libin P.J., Simmonds P., Nowé A., Muñoz-Jordán J., Alcantara L.C.J., Vandamme A.-M., Santiago G.A., Theys K. (2018). Time to harmonize dengue Nomenclature and classification. Viruses.

[2] Hung T.M., Shepard D.S., Bettis A.A., Nguyen H.A., McBride A., Clapham H.E., Turner H.C. (2020). Productivity costs from a dengue episode in Asia: A systematic literature review. BMC Infect. Dis..

[3] Hwang E.-H., Kim G., Oh H., An Y.J., Kim J., Kim J.H., Hwang E.-S., Park J.-H., Hong J., Koo B.-S. (2020). Molecular and evolutionary analysis of dengue virus serotype 2 isolates from Korean travelers in 2015. Arch. Virol..

[4] Murugesan A., Manoharan M. (2020). Dengue Virus. Emerging and Reemerging Viral Pathogens.

[5] Lozach P.-Y., Burleigh L., Staropoli I., Navarro-Sanchez E., Harriague J., Virelizier J.-L., Rey F.A., Desprès P., Arenzana-Seisdedos F., Amara A. (2005). Dendritic cell-specific intercellular adhesion molecule 3-grabbing non-integrin (DC-SIGN)-mediated enhancement of dengue virus infection is independent of DC-SIGN internalization signals. J. Biol. Chem..

[6] Carro A.C., Piccini L.E., Damonte E.B. (2018). Blockade of dengue virus entry into myeloid cells by endocytic inhibitors in the presence or absence of antibodies. PLoS Negl. Tropical. Dis..

[7] Marchiori E., Hochhegger B., Zanetti G. (2020). Pulmonary manifestations of dengue. J. Bras. Pneumol..

[8] World Health Organization (2014). Dengue and Severe Dengue.

[9] Zonetti L.F., Coutinho M.C., de Araujo A.S., Letters P. (2018). Molecular aspects of the dengue virus infection process: A review. Protein Pept. Lett..

[10] Singh A., Bisht P., Bhattacharya S., Guchhait P. (2020). Role of platelet cytokines in Dengue virus infection. Front. Cell Infect. Microbiol..

[11] Masri M.F.B., Rathore A.P., John A.L.S. (2019). Therapeutics for Dengue. Curr. Treat. Options Infect. Dis..

[12] Low J.G., Ooi E.E., Vasudevan S.G. (2017). Current status of dengue therapeutics research and development. J. Infect. Dis..

[13] Jasamai M., Yap W.B., Sakulpanich A., Jaleel A. (2019). Current prevention and potential treatment options for dengue infection. J. Pharm. Pharm. Sci..

[14] Wilder-Smith A. (2020). Dengue vaccine development by the year 2020: Challenges and prospects. Curr. Opin. Virol..

[15] Thomas S.J., Yoon I.-K. (2019). A review of Dengvaxia®: Development to deployment. Hum. Vaccin Immunother..

[16] Powers C.N., Setzer W.N. (2016). An in-silico investigation of phytochemicals as antiviral agents against dengue fever. Comb. Chem High. Throughput Screen..

[17] Sivaraman D., Pradeep P.S. (2020). Exploration of bioflavonoids targeting dengue virus NS5 RNA-dependent RNA polymerase: In silico molecular docking approach. J. App. Pharm. Sci..

[18] Tricou V., Minh N.N., Van T.P., Lee S.J., Farrar J., Wills B., Tran H.T., Simmons C.P. (2010). A randomized controlled trial of chloroquine for the treatment of dengue in Vietnamese adults. PLos Negl. Trop. Dis..

[19] Nguyen N.M., Tran C.N.B., Phung L.K., Duong K.T.H., Huynh H.l.A., Farrar J., Nguyen Q.T.H., Tran H.T., Nguyen C.V.V., Merson L. (2013). A randomized, double-blind placebo controlled trial of balapiravir, a polymerase inhibitor, in adult dengue patients. J. Infect. Dis..

[20] Dhama K., Karthik K., Khandia R., Munjal A., Tiwari R., Rana R., Khurana S.K., Ullah S., Khan R.U., Alagawany M. (2018). Medicinal and therapeutic potential of herbs and plant metabolites/extracts countering viral pathogens-current knowledge and future prospects. Curr. Drug Metab..

[21] Weinberg C.E., Weinberg Z., Hammann C. (2019). Novel ribozymes: Discovery, catalytic mechanisms, and the quest to understand biological function. Nucleic Acids Res..

[22] Tanner N.K. (1999). Ribozymes: The characteristics and properties of catalytic RNAs. FEMS Microbiol. Rev..

[23] Sczakiel G., Nedbal W. (1995). The potential of ribozymes as antiviral agents. Trends Microbiol..

[24] Menke A., Hobom G. (1997). Antiviral ribozymes. Mol. Biotechnol..

[25] Rossi J.J. (2000). Ribozyme therapy for HIV infection. Adv Drug Deliv. Rev..

[26] Lee C.H., Han S.R., Lee S.W. (2018). Therapeutic applications of group I intron-based trans-splicing ribozymes. Wiley Interdiscip Rev. RNA.

[27] Nawtaisong P., Keith J., Fraser T., Balaraman V., Kolokoltsov A., Davey R.A., Higgs S., Mohammed A., Rongsriyam Y., Komalamisra N. (2009). Effective suppression of Dengue fever virus in mosquito cell cultures using retroviral transduction of hammerhead ribozymes targeting the viral genome. Virol. J..

[28] Carter J.R., Keith J.H., Barde P.V., Fraser T.S., Fraser M.J. (2010). Targeting of highly conserved Dengue virus sequences with anti-Dengue virus trans-splicing group I introns. BMC Mol. Biol..

[29] Carter J.R., Keith J.H., Fraser T.S., Dawson J.L., Kucharski C.A., Horne K.M., Higgs S., Fraser M.J. (2014). Effective suppression of dengue virus using a novel group-I intron that induces apoptotic cell death upon infection through conditional expression of the Bax C-terminal domain. Virol. J..

[30] Carter J.R., Taylor S., Fraser T.S., Kucharski C.A., Dawson J.L., Fraser Jr M.J. (2015). Suppression of the arboviruses dengue and chikungunya using a dual-acting group-I intron coupled with conditional expression of the Bax C-terminal domain. PLoS ONE..

[31] Napoli C., Lemieux C., Jorgensen R. (1990). Introduction of a chimeric chalcone synthase gene into petunia results in reversible co-suppression of homologous genes in trans. Plant. Cell..

[32] Sen G.L., Blau H.M. (2006). A brief history of RNAi: The silence of the genes. FASEB J..

[33] Qureshi A., Tantray V.G., Kirmani A.R., Ahangar A.G. (2018). A review on current status of antiviral siRNA. Rev. Med. Virol..

[34] Lam J.K., Chow M.Y., Zhang Y., Leung S.W. (2015). siRNA versus miRNA as therapeutics for gene silencing. Mol. Ther. Nucleic Acids..

[35] Idrees S., Ashfaq U.A. (2013). RNAi: Antiviral therapy against dengue virus. Asian Pac. J. Trop Biomed..

[36] Agrawal N., Dasaradhi P., Mohmmed A., Malhotra P., Bhatnagar R.K., Mukherjee S.K. (2003). RNA interference: Biology, mechanism, and applications. Microbiol. Mol. Biol. Rev..

[37] Parashar D., Rajendran V., Shukla R., Sistla R. (2020). Lipid-based nanocarriers for delivery of small interfering RNA for therapeutic use. Eur. J. Pharm. Sci..

[38] Zhang W., Singam R., Hellermann G., Kong X., San Juan H., Lockey R.F., Wu S.-J., Porter K., Mohapatra S.S. (2004). Attenuation of dengue virus infection by adeno-associated virus-mediated siRNA delivery. Genet. Vaccines Ther..

[39] Subramanya S., Kim S.-S., Abraham S., Yao J., Kumar M., Kumar P., Haridas V., Lee S.-K., Shultz L.D., Greiner D. (2010). Targeted delivery of small interfering RNA to human dendritic cells to suppress dengue virus infection and associated proinflammatory cytokine production. J Virol..

[40] Yue J., Wu X., Wu Y., Li X., Jiang L., Li Q., Li L., Yang X. (2010). Study on the inhibitory effect of RNA interference on replication of dengue virus. Bing Du Xue Bao..

[41] Stein D.A., Perry S.T., Buck M.D., Oehmen C.S., Fischer M.A., Poore E., Smith J.L., Lancaster A.M., Hirsch A.J., Slifka M.K. (2011). Inhibition of dengue virus infections in cell cultures and in AG129 mice by a small interfering RNA targeting a highly conserved sequence. J. Virol..

[42] Ang F., Wong A.P.Y., Ng M.M.-L., Chu J.J.H. (2010). Small interference RNA profiling reveals the essential role of human membrane trafficking genes in mediating the infectious entry of dengue virus. Virol. J..

[43] Alhoot M.A., Wang S.M., Sekaran S.D. (2012). RNA interference mediated inhibition of dengue virus multiplication and entry in HepG2 cells. PLoS ONE..

[44] Villegas-Rosales P.M., Méndez-Tenorio A., Ortega-Soto E., Barrón B.L. (2012). Bioinformatics prediction of siRNAs as potential antiviral agents against dengue viruses. Bioinformation..

[45] Villegas P.M., Ortega E., Villa-Tanaca L., Barrón B.L., Torres-Flores J. (2018). Inhibition of dengue virus infection by small interfering RNAs that target highly conserved sequences in the NS4B or NS5 coding regions. Arch Virol..

[46] Yan H., Zhou Y., Liu Y., Deng Y., Puthiyakunnon S., Chen X. (2014). MiR-252 of the Asian tiger mosquito Aedes albopictus regulates dengue virus replication by suppressing the expression of the dengue virus envelope protein. J Med. Virol..

[47] Wen W., He Z., Jing Q., Hu Y., Lin C., Zhou R., Wang X., Su Y., Yuan J., Chen Z. (2015). Cellular microRNA-miR-548g-3p modulates the replication of dengue virus. J. Infect..

[48] Castillo J.A., Castrillón J.C., Diosa-Toro M., Betancur J.G., St Laurent G., Smit J.M., Urcuqui-Inchima S.J.B.i.d. (2015). Complex interaction between dengue virus replication and expression of miRNA-133a. BMC Inf Dis..

[49] Castrillón-Betancur J.C., Urcuqui-Inchima S. (2017). Overexpression of miR-484 and miR-744 in Vero cells alters Dengue virus replication. Mem Inst Oswaldo Cruz..

[50] Ellington A.D., Szostak J.W. (1990). In vitro selection of RNA molecules that bind specific ligands. Nature.

[51] Tuerk C., Gold L. (1990). Systematic evolution of ligands by exponential enrichment: RNA ligands to bacteriophage T4 DNA polymerase. Science..

[52] Berzal-Herranz A., Romero-López C. (2020). Two Examples of RNA Aptamers with Antiviral Activity. Are Aptamers the Wished Antiviral Drugs?. Pharmaceuticals.

[53] González V.M., Martín M.E., Fernández G., García-Sacristán A. (2016). Use of aptamers as diagnostics tools and antiviral agents for human viruses. Pharmaceuticals.

[54] Kedzierski S., Khoshnejad M., Caltagirone G.T. (2012). Synthetic antibodies: The emerging field of aptamers. BioProcess J..

[55] Bouchard P., Hutabarat R., Thompson K. (2010). Discovery and development of therapeutic aptamers. Annu. Rev. Pharmacol. Toxicol..

[56] Marton S., Reyes-Darias J.A., Sánchez-Luque F.J., Romero-López C., Berzal-Herranz A. (2010). In vitro and ex vivo selection procedures for identifying potentially therapeutic DNA and RNA molecules. Molecules.

[57] Kulkarni R. (2020). Antibody-Dependent Enhancement of Viral Infections. Dynamics of Immune Activation in Viral Diseases.

[58] Balinsky C.A., Schmeisser H., Ganesan S., Singh K., Pierson T.C., Zoon K.C. (2013). Nucleolin interacts with the dengue virus capsid protein and plays a role in formation of infectious virus particles. J. Virol..

[59] Chen H.-L., Hsiao W.-H., Lee H.-C., Wu S.-C., Cheng J.-W. (2015). Selection and characterization of DNA aptamers targeting all four serotypes of dengue viruses. PLoS ONE..

[60] Jung J.I., Han S.R., Lee S.-W. (2018). Development of RNA aptamer that inhibits methyltransferase activity of dengue virus. Biotechnol Lett..

[61] Summerton J.E. (2017). Invention and early history of morpholinos: From pipe dream to practical products. Morpholino Oligomers. Methods in Molecular Biology.

[62] Mendell J.R., Rodino-Klapac L.R., Sahenk Z., Roush K., Bird L., Lowes L.P., Alfano L., Gomez A.M., Lewis S., Kota J. (2013). Eteplirsen for the treatment of Duchenne muscular dystrophy. Ann. Neurol..

[63] Summerton J., Weller D. (1997). Morpholino antisense oligomers: Design, preparation, and properties. Antisense Nucleic Acid Drug Dev..

[64] Morcos P.A., Li Y., Jiang S. (2008). Vivo-Morpholinos: A non-peptide transporter delivers Morpholinos into a wide array of mouse tissues. Biotechniques.

[65] Nan Y., Zhang Y.-J. (2018). Antisense phosphorodiamidate morpholino oligomers as novel antiviral compounds. Front. Microbiol..

[66] Kinney R.M., Huang C.Y.-H., Rose B.C., Kroeker A.D., Dreher T.W., Iversen P.L., Stein D.A. (2005). Inhibition of dengue virus serotypes 1 to 4 in vero cell cultures with morpholino oligomers. J. Virol..

[67] Holden K.L., Stein D.A., Pierson T.C., Ahmed A.A., Clyde K., Iversen P.L., Harris E. (2006). Inhibition of dengue virus translation and RNA synthesis by a morpholino oligomer targeted to the top of the terminal 3′ stem–loop structure. Virology.

[68] Stein D.A., Huang C.Y.-H., Silengo S., Amantana A., Crumley S., Blouch R.E., Iversen P.L., Kinney R.M. (2008). Treatment of AG129 mice with antisense morpholino oligomers increases survival time following challenge with dengue 2 virus. J. Antimicrob Chemother..

[69] Phumesin P., Junking M., Panya A., Yongpitakwattana P., Noisakran S., Limjindaporn T., Yenchitsomanus P.-T. (2018). Vivo-morpholino oligomers strongly inhibit dengue virus replication and production. Arch. Virol..

[70] Phumesin P., Junking M., Panya A., Yongpitakwattana P., Noisakran S., Limjindaporn T., Yenchitsomanus P.-T. (2019). Inhibition of dengue virus replication in monocyte-derived dendritic cells by vivo-morpholino oligomers. Virus Res..

[71] Bayat H., Naderi F., Khan A.H., Memarnejadian A., Rahimpour A. (2018). The impact of crispr-cas system on antiviral therapy. Adv. Pharm Bull..

[72] Zhang R., Miner J.J., Gorman M.J., Rausch K., Ramage H., White J.P., Zuiani A., Zhang P., Fernandez E., Zhang Q. (2016). A CRISPR screen defines a signal peptide processing pathway required by flaviviruses. Nature.

[73] Savidis G., McDougall W.M., Meraner P., Perreira J.M., Portmann J.M., Trincucci G., John S.P., Aker A.M., Renzette N., Robbins D.R. (2016). Identification of Zika virus and dengue virus dependency factors using functional genomics. Cell Rep..

[74] Marceau C.D., Puschnik A.S., Majzoub K., Ooi Y.S., Brewer S.M., Fuchs G., Swaminathan K., Mata M.A., Elias J.E., Sarnow P. (2016). Genetic dissection of Flaviviridae host factors through genome-scale CRISPR screens. Nature.

[75] Abudayyeh O.O., Gootenberg J.S., Essletzbichler P., Han S., Joung J., Belanto J.J., Verdine V., Cox D.B., Kellner M.J., Regev A. (2017). RNA targeting with CRISPR–Cas13. Nature.

[76] Kumar N., Marx D. (2020). Deciphering the Self-Cleavage Reaction Mechanism of Hairpin Ribozyme. J. Phys. Chem B..

[77] Xu W., Jiang X., Huang L. (2019). RNA Interference Technology. Compr. Biotechnol..

[78] Buglak A.A., Samokhvalov A.V., Zherdev A.V., Dzantiev B.B. (2020). Methods and Applications of In Silico Aptamer Design and Modeling. Int. J. Mol. Sci..

[79] Roberts T.C., Langer R., Wood M.J. (2020). Advances in oligonucleotide drug delivery. Nat. Rev. Drug Discov..

[80] Perreault J., Weinberg Z., Roth A., Popescu O., Chartrand P., Ferbeyre G., Breaker R.R. (2011). Identification of hammerhead ribozymes in all domains of life reveals novel structural variations. PLoS Comput Biol..

[81] Hedberg A., Johansen S.D. (2013). Nuclear group I introns in self-splicing and beyond. Mob. DNA..

[82] Svoboda P. (2020). Key Mechanistic Principles and Considerations Concerning RNA Interference. Front. Plant. Sci..

[83] Zhang Y., Lai B.S., Juhas M. (2019). Recent advances in aptamer discovery and applications. Molecules.

[84] Quijano E., Bahal R., Ricciardi A., Saltzman W.M., Glazer P.M. (2017). Therapeutic Peptide Nucleic Acids: Principles, Limitations, and Opportunities. Yale J. Biol. Med..

[85] Hertel K.J., Herschlag D., Uhlenbeck O.C. (1996). Specificity of hammerhead ribozyme cleavage. EMBO J..

[86] Kalra P., Dhiman A., Cho W.C., Bruno J.G., Sharma T.K. (2018). Simple methods and rational design for enhancing aptamer sensitivity and specificity. Front. Mol. Biosci..

[87] Blum M., De Robertis E.M., Wallingford J.B., Niehrs C. (2015). Morpholinos: Antisense and sensibility. Dev. Cell..

[88] Kharma N., Varin L., Abu-Baker A., Ouellet J., Najeh S., Ehdaeivand M.-R., Belmonte G., Ambri A., Rouleau G., Perreault J. (2016). Automated design of hammerhead ribozymes and validation by targeting the PABPN1 gene transcript. Nucleic Acids Res..

[89] Bartoszewski R., Sikorski A.F. (2019). Editorial focus: Understanding off-target effects as the key to successful RNAi therapy. Cell Mol. Biol. Lett..

[90] Zhu G., Chen X. (2018). Aptamer-based targeted therapy. Adv. Drug Deliv. Rev..

[91] Eisen J.S., Smith J.C. (2008). Controlling morpholino experiments: Don’t stop making antisense. Development.

[92] Chu C.-Y., Rana T.M. (2008). Potent RNAi by short RNA triggers. RNA.

[93] Zou X., Wu J., Gu J., Shen L., Mao L. (2019). Application of aptamers in virus detection and antiviral therapy. Front. Microbiol..

[94] Moulton J.D., Yan Y.L. (2008). Using Morpholinos to control gene expression. Curr. Protoc. Mol. Biol..

[95] Westhof E., Fritsch V. (2000). RNA folding: Beyond Watson–Crick pairs. Structure.

[96] Kim D.H., Rossi J.J. (2008). RNAi mechanisms and applications. Biotechniques.

[97] Hidding J. (2016). A therapeutic battle: Antibodies vs. Aptamers. Nanosci. Master Program..

[98] Xiao G., Wesolowski D., Izadjoo M., Altman S. (2010). Morpholino oligonucleotides do not participate perfectly in standard Watson-Crick complexes with RNA. RNA.

[99] Delley C.L., Liu L., Sarhan M.F., Abate A.R. (2018). Combined aptamer and transcriptome sequencing of single cells. Sci. Rep..

[100] Bedell V.M., Westcot S.E., Ekker S.C. (2011). Lessons from morpholino-based screening in zebrafish. Brief. Funct Genomics..

[101] Lewin A.S., Hauswirth W.W. (2001). Ribozyme gene therapy: Applications for molecular medicine. Trends Mol. Med..

[102] Heigwer F., Port F., Boutros M. (2018). RNA interference (RNAi) screening in Drosophila. Genetics.

[103] Esposito C.L., Catuogno S., Condorelli G., Ungaro P., De Franciscis V. (2018). Aptamer chimeras for therapeutic delivery: The challenging perspectives. Genes (Basel)..

[104] Lakhin A., Tarantul V., Gening L. (2013). Aptamers: Problems, solutions and prospects. Acta Naturae..

[105] Burnett J.C., Rossi J.J. (2012). RNA-based therapeutics: Current progress and future prospects. Chem. Biol..

[106] Ovcharenko D., Jarvis R., Hunicke-Smith S., Kelnar K., Brown D. (2005). High-throughput RNAi screening in vitro: From cell lines to primary cells. RNA.

[107] Scott W.G. (2007). Ribozymes. Curr. Opin. Struct Biol..

[108] Joga M.R., Zotti M.J., Smagghe G., Christiaens O. (2016). RNAi efficiency, systemic properties, and novel delivery methods for pest insect control: What we know so far. Front. Physiol..

[109] Phylactou L.A., Kilpatrick M.W., Wood M.J. (1998). Ribozymes as therapeutic tools for genetic disease. Hum. Mol. Genet..

[110] Berkhout B. (2018). RNAi-mediated antiviral immunity in mammals. Curr. Opin. Virol..

[111] Song K.-M., Lee S., Ban C. (2012). Aptamers and their biological applications. Sensors.

[112] Pandey S.N., Lee Y.-C., Yokota T., Chen Y.-W. (2014). Morpholino treatment improves muscle function and pathology of Pitx1 transgenic mice. Mol. Ther..

[113] Gurav B., Srinivasan G. (2017). Antisense oligonucleotides as therapeutics and their delivery. Curr. Sci..

[114] Da Pieve C., Blackshaw E., Missailidis S., Perkins A.C. (2012). PEGylation and biodistribution of an anti-MUC1 aptamer in MCF-7 tumor-bearing mice. Bioconjug Chem..

[115] Deas T.S., Bennett C.J., Jones S.A., Tilgner M., Ren P., Behr M.J., Stein D.A., Iversen P.L., Kramer L.D., Bernard K.A. (2007). In vitro resistance selection and in vivo efficacy of morpholino oligomers against West Nile virus. Antimicrob. Agents Chemother..

[116] Kugelman J.R., Sanchez-Lockhart M., Andersen K.G., Gire S., Park D.J., Sealfon R., Lin A.E., Wohl S., Sabeti P.C., Kuhn J.H. (2015). Evaluation of the potential impact of Ebola virus genomic drift on the efficacy of sequence-based candidate therapeutics. mBio.

[117] Seelam Prabhakar P., Manderville R.A., Wetmore S.D. (2019). Impact of the Position of the Chemically Modified 5-Furyl-2′-Deoxyuridine Nucleoside on the Thrombin DNA Aptamer–Protein Complex: Structural Insights into Aptamer Response from MD Simulations. Molecules.

[118] Seley-Radtke K. (2020). Discovery, Design, Synthesis, and Application of Nucleoside/Nucleotides. Molecules.

[119] Alves Ferreira-Bravo I., Cozens C., Holliger P., DeStefano J.J. (2015). Selection of 2′-deoxy-2′-fluoroarabinonucleotide (FANA) aptamers that bind HIV-1 reverse transcriptase with picomolar affinity. Nucleic Acids Res..

[120] Eremeeva E., Fikatas A., Margamuljana L., Abramov M., Schols D., Groaz E., Herdewijn P. (2019). Highly stable hexitol based XNA aptamers targeting the vascular endothelial growth factor. Nucleic Acids Res..

[121] Taylor A.I., Houlihan G., Holliger P. (2019). Beyond DNA and RNA: The expanding toolbox of synthetic genetics. Cold Spring Harb Perspect Biol..

